# Supplementary material for: A Bayesian Hierarchical Model of Trial-To-Trial Fluctuations in Decision Criterion
Source: bioRxiv. 2024 Oct 2:2024.07.30.605869. Originally published 2024 Jul 31. Preprint. [Version 2] doi: 10.1101/2024.07.30.605869 (PMC11361103; doi:10.1101/2024.07.30.605869)
Supplement: 1 [file NIHPP2024.07.30.605869V2-supplement-1.pdf]

bioRxiv preprint doi: https://doi.org/10.1101/2024.07.30.605869; this version posted October 2, 2024. The copyright holder for this preprint (which was not certified by peer review) is the author/funder, who has granted bioRxiv a license to display the preprint in perpetuity. It is made available under aCC-BY 4.0 International license.

638 Supplemental Figures

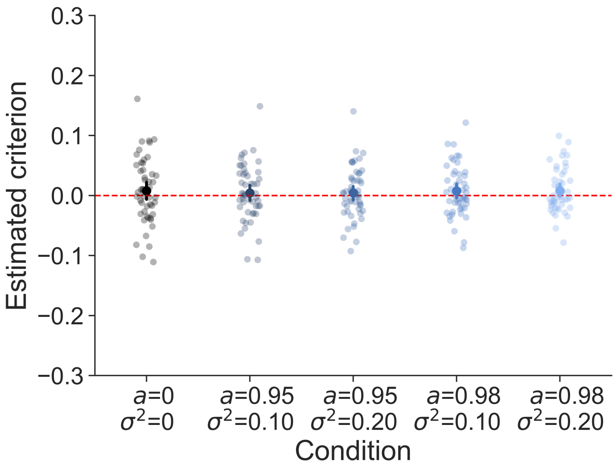

Figure S1: Whereas the presence of criterion fluctuations leads to an underestimation of d', a popular signal-detection measure of sensitivity (fig. 1E), the criterion is on average correctly estimated when using the traditional method assuming a stable criterion. The red line shows the true criterion value.

bioRxiv preprint doi: https://doi.org/10.1101/2024.07.30.605869; this version posted October 2, 2024. The copyright holder for this preprint (which was not certified by peer review) is the author/funder, who has granted bioRxiv a license to display the preprint in perpetuity. It is made available under aCC-BY 4.0 International license.

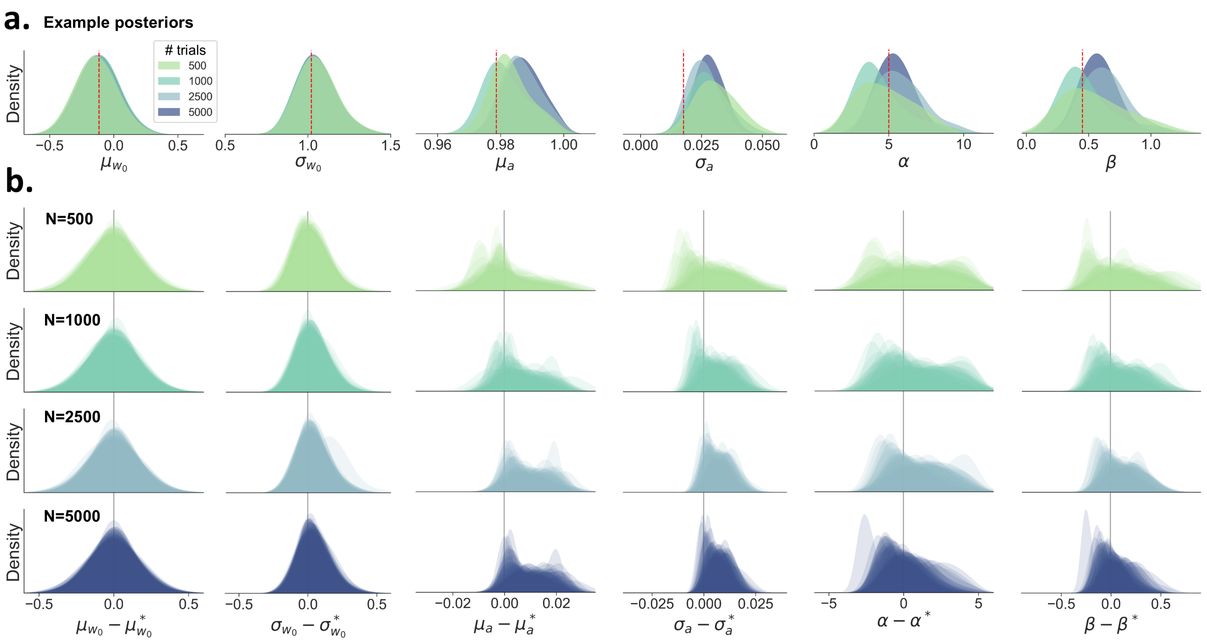

Figure S2: The recovery of the group-level (global) parameters in function of the number of trials per subject (500, 1000, 2500, 5000). In total, 50 datasets were simulated, each with 50 subjects. A) An example posterior is shown for each parameter and subject level. In contrast to fig. 3, where the posteriors become more narrow when increasing the number of subjects, here we see that the posterior precision remains the same with increasing trial counts. B) The overlaid posteriors are shown for all 50 datasets. The estimated posteriors are corrected and centered on the true value (denoted by an asterisk), similar to fig. 3.

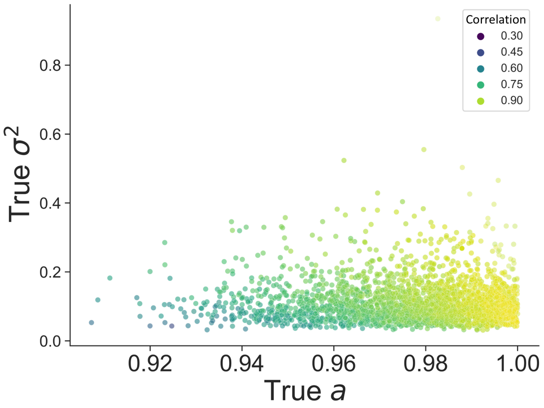

Figure S3: The correlation between the true and the estimated criterion trajectory in function of the true values for a and σ^2. The criterion trajectory is harder to recover when lower values for a are combined with lower values for σ^2. These parameter values typically generate time series that do not fluctuate much and overall stay relatively close to its baseline, making it harder for the model to identify.
